# Supplementary material for: Surface Modification by Nano-Structures Reduces Viable Bacterial Biofilm in Aerobic and Anaerobic Environments
Source: Int J Mol Sci. 2020 Oct 6;21(19):7370. doi: 10.3390/ijms21197370 (PMC7582899; doi:10.3390/ijms21197370)
Supplement: Supplementary file 1 [file ijms-21-07370-s001.pdf]

Supporting Information

Surface Modification by Nano-Structures Reduces Viable Bacterial Biofilm in Aerobic and Anaerobic Environments

Sarah Ya'ari [1,2,3], Michal Halperin-Sternfeld [1,2,3], Boris Rosin [1,2,3] and Lihi Adler-Abramovich [1,2,3,*]

1 Department of Oral Biology, The Goldschleger School of Dental Medicine, Sackler Faculty of Medicine, Tel Aviv University, Tel Aviv 6997801, Israel; sarahkal@gmail.com (S.Y.); michal4@mail.tau.ac.il (M.H.-S.); borisrosin@mail.tau.ac.il (B.R.)

2 The Center for Nanoscience and Nanotechnology, Tel Aviv University, Tel Aviv 6997801, Israel

3 The Center for the Physics and Chemistry of Living Systems, Tel Aviv University, Tel Aviv 6997801, Israel

* Correspondence: LihiA@tauex.tau.ac.il; Tel.: +972-3-640-7252

Keywords: modified amino acid; self-assembly; surface coating; anti-biofilm activity

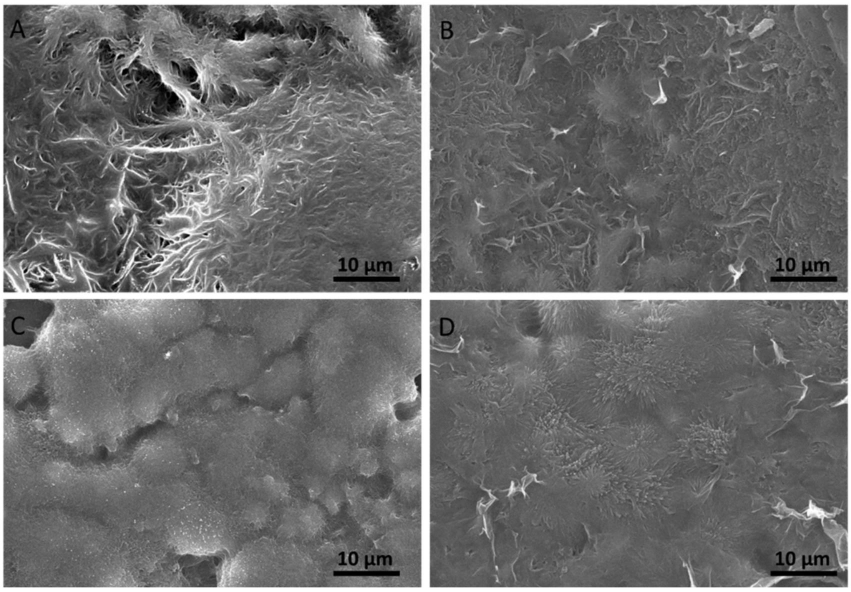

Figure S1. SEM images of glass and mica surfaces coated with Fmoc-F_5-Phe. (**A**-**B**) SEM images of Fmoc-F_5-Phe modified (**A**) glass and (**B**) mica before stability test. (**C**-**D**) SEM images of Fmoc-F_5-Phe modified (**C**) glass and (**D**) mica after stability test.

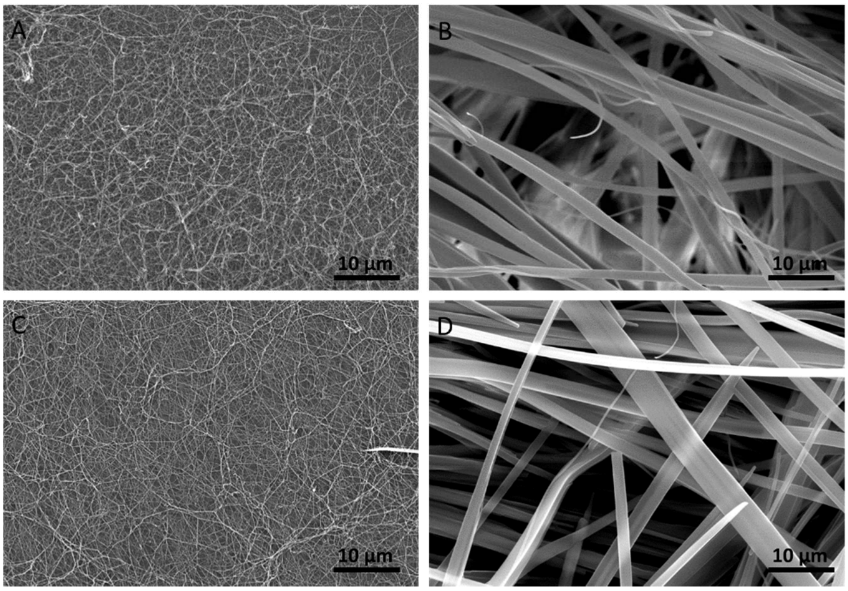

Figure S2. SEM images of glass and mica surfaces coated with Boc-F_5-Phe. (**A**-**B**) SEM images of Boc-F_5-Phe modified (**A**) glass and (**B**) mica before stability test. (**C**-**D**) SEM images of Boc-F_5-Phe modified (**C**) glass and (**D**) mica after stability test.

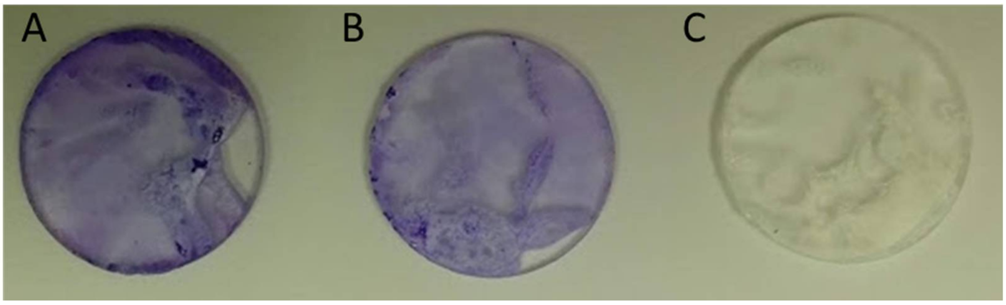

Figure S3. Initial biofilm analysis for siliconized glass coated with Fmoc-F_5-Phe. (**A**) Fmoc-F_5-Phe modified slides without bacteria stained with crystal violet (**B**) Fmoc-F_5-Phe stained with crystal violet and washed overnight and (**C**) non-stained control sample.

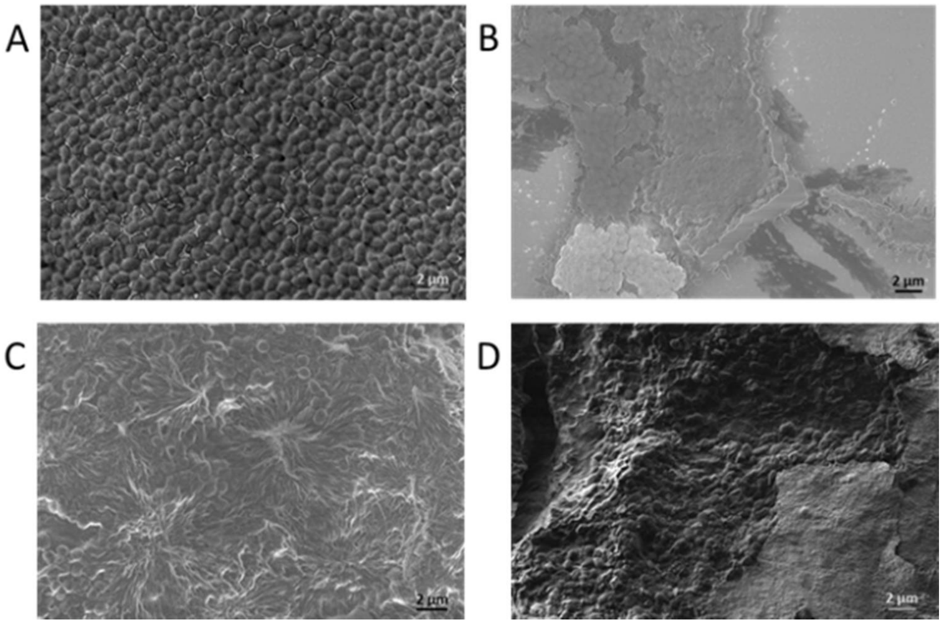

Figure S4. Biofilm analysis by HRSEM. (**A**) *E. faecalis* and (**B**) *S. mutans* form biofilm on non-coated surface, (**C**) *E. faecalis* and (**D**) *S. mutans* incubated on Fmoc-F_5-Phe coated surface.
